# Representation control increases task efficiency in complex graphical representations

**DOI:** 10.1371/journal.pone.0196420

**Published:** 2018-04-26

**Authors:** Julia Moritz, Hauke S. Meyerhoff, Claudia Meyer-Dernbecher, Stephan Schwan

**Affiliations:** Leibniz-Institut für Wissensmedien, Tübingen, Germany; Fordham University, UNITED STATES

## Abstract

In complex graphical representations, the relevant information for a specific task is often distributed across multiple spatial locations. In such situations, understanding the representation requires internal transformation processes in order to extract the relevant information. However, digital technology enables observers to alter the spatial arrangement of depicted information and therefore to offload the transformation processes. The objective of this study was to investigate the use of such a representation control (i.e. the users' option to decide how information should be displayed) in order to accomplish an information extraction task in terms of solution time and accuracy. In the representation control condition, the participants were allowed to reorganize the graphical representation and reduce information density. In the control condition, no interactive features were offered. We observed that participants in the representation control condition solved tasks that required reorganization of the maps faster and more accurate than participants without representation control. The present findings demonstrate how processes of cognitive offloading, spatial contiguity, and information coherence interact in knowledge media intended for broad and diverse groups of recipients.

## Introduction

Complex graphical representations such as depictions of the results of elections frequently occur in traditional as well as online media [1,2]. While complex graphical representations appear in many different forms, they all share a spatial organization of visual/pictorial and verbal information. However, as goals and information needs vary between different observers, complex graphical representations typically include unnecessary information that needs to be ignored. For instance, consider a graphical representation depicting the development of state elections across two election periods. When one observer is interested only in the outcome of the current elections, he or she needs to ignore all information related to previous elections, whereas a second observer who is interested in the change between the last and the present election needs to attend to the full information. In the present study, we investigated whether representation control (i.e. the ability to alter the representation of the displayed information) enhances the efficiency as well as the accuracy of extracting relevant information from complex graphical representations.

For printed media, designers of graphical representations face a conflict between ease of use and usefulness: When the viewers' information task is known in advance, the graphical representation can be tailored accordingly by leaving out task-irrelevant information and focusing on task-relevant information [3,4]. The graphical representation may then be kept simple, maximizing ease of use, but on the other hand, the usefulness of the graphical representation is restricted to only one specific task. Alternatively, designers may choose to include more information in the graphical representation than is necessary for one particular task. In this case, the graphical representation is suitable for a broader range of tasks/questions. Consequently, the usefulness of the graphical representation is maximized, but information may be cluttered, impeding the extraction of the information relevant for a specific task. In return, this restricts the ease of use.

Digital graphical representations (e.g., in online media) offer a solution to mediate this conflict between ease of use and usefulness; designers may include information suitable for a wide range of tasks and allow the viewers themselves to interactively adapt a given graphical representation to their individual needs and task requirements. While such interactive graphical representations seem to combine both a high degree of ease of use and usefulness, adapting graphical representations requires metacognitive knowledge as well as task-specific knowledge about the structure of the current task. Furthermore, a new tradeoff arises between the motoric and cognitive costs of altering the depicted information and the reduced cognitive effort that is necessary to subsequently comprehend the altered information (see also [5]).

Tradeoffs between costs of motoric actions and cognitive effort are not unique for graphical representations but provide a rather broad principle of cognitive psychology. In fact, even actions such as writing notes or calendar entries can be considered to be cognitive offloading in order to relieve internal processing capacities. This externalization of internal processes is referred to as cognitive offloading [6,7] that is defined as “the use of physical action to alter the information processing requirements of a task so as to reduce cognitive demand” [8]. Scaife & Rogers [9] identified computational offloading as one of three central characteristics of using external representations for problem solving (besides re-representation and graphical constraining). They also assume that the use of graphical representations reduces cognitive effort in problem solving. In a similar vein, in information visualization research, three purposes of physical actions on data visualizations were identified: external anchoring for coupling of internal and external representations, information foraging, and cognitive offloading of memory [10]. Remarkably, the externalization processes of cognitive offloading are not restricted to memory representations, but also include physical transformations that aim at avoiding corresponding mental operations [11]. For instance, Kirsh and Maglio [12] demonstrated that participants rather perform motoric actions that alter the representation of information in the environment (i.e. rotating Tetris blocks) than simulating the outcome of the motoric action mentally (i.e. corresponding mental rotation). However, it is unclear whether in complex graphical representations such externalizations of transformation processes are executed, as the representations involve semantic meaning and combine multiple representation formats. The transformation of complex information requires more knowledge about the exact type and organization of the required information, about the task structure, and about the strategy how to achieve an optimal information representation. Thus, requirements—such as metacognitive knowledge—for cognitive offloading in interactive graphical representations are higher than in the above-mentioned studies. In the present study, we tested whether the principles of cognitive offloading also apply to the usage of interactive features in these complex graphical representations.

With regard to information complexity, infographics clearly exceed the materials of previous studies on cognitive offloading. These studies used Tetris blocks [12] or colored squares in the pattern copy task for investigating the cognitive offloading of mental rotation or memory [7,13,14]. Others used rotated text displays [15], sets of letters [16,17] or simple geometrical figures [17,18]. Successful offloading of cognitive processes on infographics requires an adequate adaptation of the depicted information to the task at hand. Representation control allows the user to adapt the representational format to the task requirements [19,20]. With regard to cognitive offloading, representation control allows for the externalization of the spatial transformation as well as the selection of depicted information. In our study, these two fundamental transformations were enabled as part of representation control: (a) changing the spatial logic of the graphical representation, that is, the meaning of distances between different elements (i.e. spatial organization), and (b) reducing information density according to the relevance of information (i.e. selection). As the selection does not influence the spatial layout of the infographic, a reduction of information results in lower information density. These transformations were selected because they are based on well-studied principles of cognitive processing of multimedia [21–24] as well as practical advice [3,25].

The change of spatial organization affects the spatial contiguity (i.e. spatial proximity) of the depicted information [21–24,26]. Studies on learning from text and pictures showed enhanced learning performance when text and picture are integrated rather than presented separately [27]. An explanation for this effect is that search and integration of corresponding pieces of information require additional cognitive processing. These resources then are missing for elaboration and knowledge transfer [28], resulting in lower performance in des-integrated formats. Studies that have investigated the cognitive processes in multimedia learning through eye tracking have additionally demonstrated that participants make fewer eye movements connecting the corresponding information units in split formats or even ignored information units [29,30]. An advantage of integrated presentation formats is found not only in learning tasks but also in visual comparison tasks. Higher working memory use in trials with larger distances explains this effect [31,32]. However, optimal spatial organizations vary between different tasks. In other words, different tasks may require different information elements to be presented close to each other. Therefore, while the fixed layout of a graphical representation may foster solving a particular task, it may be detrimental for a range of different tasks. As a possible solution, providing representation control comes into play in order to allow for switching between different spatial organizations of the graphical representation.

The second transformation (selection) is based on the distinction between relevant and irrelevant information. According to the coherence principle of the cognitive theory of multimedia learning [22], simpler displays are more beneficial for learning. Two solutions for indicating this distinction between relevant and irrelevant information were proposed: the highlighting of relevant information and the removal of irrelevant information [33]. Studies on weather maps underpin the effectiveness of these two solutions. Regarding highlighting, studies have shown an influence of information salience on information processing [34–36]. Similarly, experimental conditions with task-irrelevant information impaired performance relative to conditions without irrelevant information [37]. Furthermore, irrelevant information included in maps led to longer processing time and less accurate responses [4]. Remarkably, this advantage of the reduction to relevant information stands in contrast to preferences for realistic and thus complex displays. Map users tend to choose more realistic and complex maps over abstract and reduced ones [4,38,39].

In the present study, we investigated how representation control contributes to cognitive offloading as well as task performance, using complex graphical representations as stimuli. We asked participants to perform an information extraction task with political maps on which the relevant information was either spatially aligned (match) or misaligned (mismatch) with the task demands. In our experiment, we compared offloading as well as performance between two groups that were either allowed to alter the graphical representation (i.e. representation control) or not (static condition). We hypothesized that when available, representation control will be used in a task-appropriate manner to increase task effectiveness. More specifically, we predicted:

(a) Users provided with representation control will adjust the complex graphical representations in cases in which the overall spatial organization does not correspond to the task requirements.
(b) Users with representation control will reduce the information density of the complex graphical representations in order to increase coherence of the depicted information.
(c) Users with representation control will be faster in the information extraction tasks with (initially) mismatching graphical representations than users in the static condition.
(d) Users with representation control will be more accurate than users in the static condition.

## Methods

### Participants

The final sample consisted of 88 participants (66 female; mean age: 27.5 years, range 19–63 years) who were recruited from the participant pool of the *Leibniz-Institut für Wissensmedien*, Tübingen. They were randomly assigned to the two experimental conditions. The experimental procedure was approved by the ethics committee of the *Leibniz-Institut für Wissensmedien*, Tübingen. Most of the participants were university students (90%) with a relatively high self-reported experience in the use of computers (on a scale from 1 (daily use)—to 4 (practically no use): *M* = 1.06; *SD* = 0.27) but low self-reported experience in the use of tablets (*M* = 3.48; *SD* = 0.94). All participants provided written informed consent prior to testing. They received monetary compensation (10 €). One participant was excluded due to self-reported color blindness. An additional participant was excluded due to no correct responses (and therefore no valid data for RT analyses) in the mismatch trials. Both participants were part of the static group.

### Materials

#### Hardware

The experiment was conducted on Microsoft Surface Pro2 tablets with a 10.6-inch screen (1920 x 1080 pixels). All user input was given through the touch-sensitive display.

#### Graphical representations

**Fig 1:**
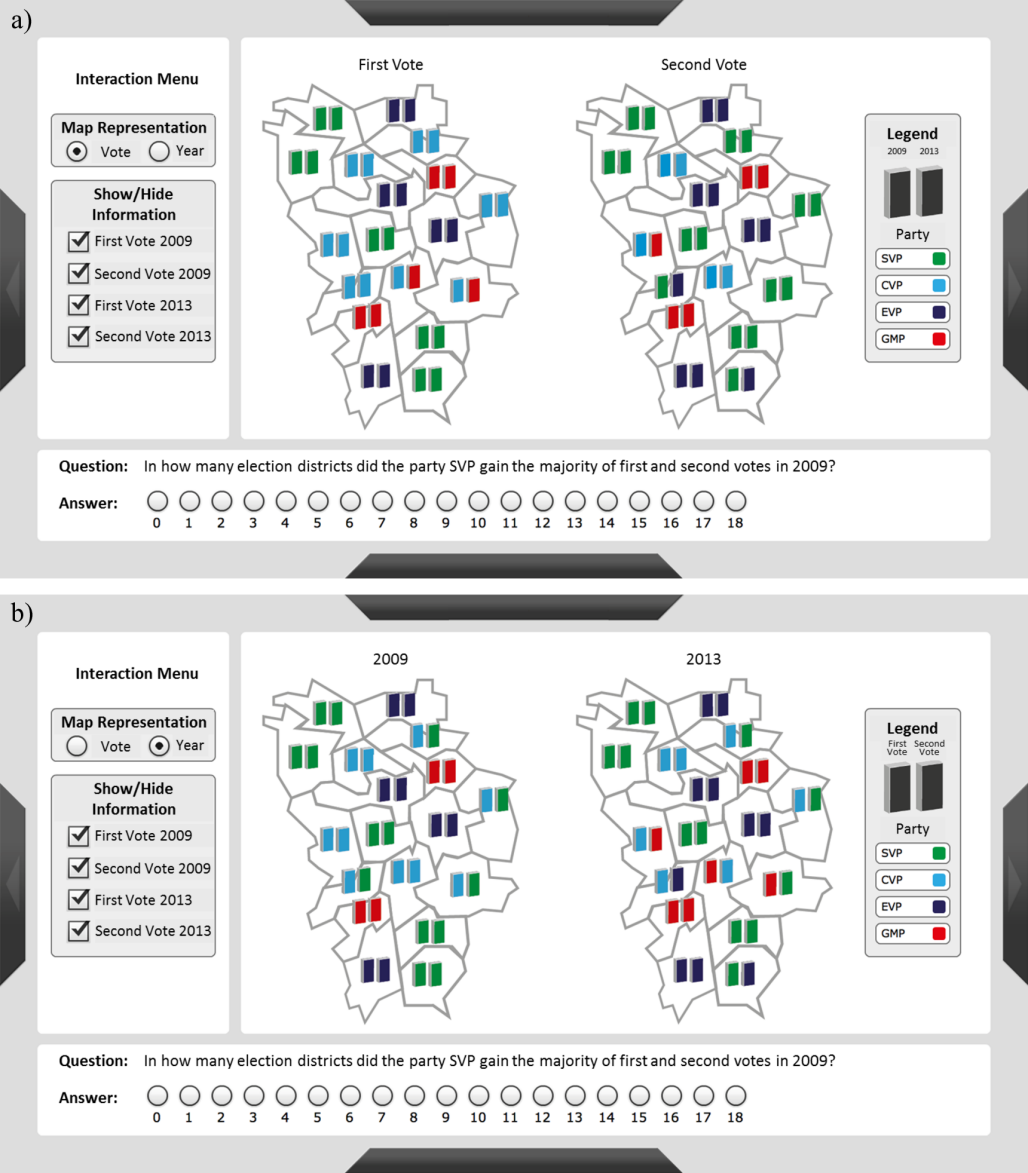
Layout and components of the graphical representation for the representation control condition. The pair of maps in the center of the screen is surrounded by the interaction menu on the left and the legend on the right. The question/answer field is located below the maps. a) The graphical representation is organized by type of vote, resulting in a mismatch trial when combined with the depicted question. b) The graphical representation is organized by election period, resulting in a match trial when combined with the depicted question.

The graphical representations included two maps that were arranged side by side and depicted fictitious election results of two consecutive election periods and two types of votes. The two types of votes, called first vote and second vote, were based on the German election system, in which the seats of the parliament are distributed according to the combined result of the two types of votes. The exact layout and the components of the display are depicted in Fig 1. Each map consisted of 18 districts with two colored bars. The bars showed the color of the winner´s party and were explained in a legend.

The depicted election results were either organized by type of vote (Fig 1A) or election period (Fig 1B). In the organization by election period, the left map showed the election results for the first election period, while the right map showed the results for the second election period. The left bars in each map represented the first votes, whereas the right bars represented the second votes. In the organization by type of vote, the left map showed the results for the first vote, and the right map showed the results for the second vote. Further, the left bar showed the winner of the vote in the first election period, and the right bar showed the winner of the second election period.

The questions in the question/answer field either focused on the two types of votes within one election period or on one type of vote across the two election periods. For instance, the question ‘In how many election districts did the party SVP gain the majority of the first votes, in both 2009 and 2013?’ required the participants to compare the first votes of the two election periods, whereas the question ‘In how many election districts did the party SVP gain the majority of the first and the second votes in 2009?’ required the participants to compare the first and the second votes within one election period. All questions were of the described format, only varying in the parties, the election periods, and the type of votes. Participants indicated their responses by marking one of the 19 answer options below the infographic.

#### Match/Mismatch trials

The combination of the two types of map organization and the two types of questions resulted in match and mismatch trials. In match trials, all information required for solving a task was visible within one map. Therefore, participants only had to identify the color of the party in question as well as the relevant map in match trials in order to subsequently count the states. In mismatch trails, however, the information required to solve a task was distributed between the two maps. Therefore, the participants also had to identify the color of the party as well as the relevant information; however, in these trials the relevant information was displayed as either the right bar or the left bar in both maps. Thus, the participants had to compare the bars in the two maps in order to count the states with consistent votes. Importantly, within the representation control condition, the participants were able to change the organization criterion of the maps. In this condition, the terms match and mismatch refer to the initial state of the maps.

### Procedure

After providing informed consent, participants received instructions explaining the information extraction task. Participants in the representation control condition watched an additional instruction video that explained the condition-specific interactive functions. We instructed the participants only how the representation control features could be used in principle; however, we did not encourage them to use these features whenever applicable. Next, the participants completed four practice trials (two match and two mismatch trials), of which three had to be answered correctly. In case of more than one error, the practice trials were repeated until the criterion was met. Following these practice trials, the participants completed 24 experimental trials. One half of the trials consisted of match trials, the other half of mismatch trails. Match and mismatch trials alternated, the order being consistent among the participants. At the beginning of each trial, all information layers (types of votes and election periods) were visible.

In the representation control condition, participants could reorganize the maps by switching the organization criterion in an interaction menu on the left side of the screen (see Fig 1). Participants could also select and deselect information layers. In the static condition, no interaction menu was provided. In this condition, the participants solved the task based on the pair of maps without the option to reorganize and/or reduce information. The participants were allowed to change their response until they confirmed it and proceeded with the next trial.

### Analysis

We analyzed response times (RTs) and error rates (ERs) as dependent variables. In the representation control group, we additionally analyzed changes to the map organization and the information density. The trials with a response time larger than four standard deviations from the mean (0.90%) were excluded from the analysis. Errors were dichotomously coded and the trials with inaccurate responses were ignored in all subsequent analyses. We used linear mixed effect models (LME) to analyze the log-transformed RTs (due to their leftward inclination). In order to analyze the ERs and layout changes, we used generalized linear mixed effect models (GLME) with the logit as link function.

Fixed effects in the RT and the ER analyses were condition (representation control vs. static), matching (match vs. mismatch), and the interaction between condition and matching. In the analysis of the changes of the layout, only the fixed effect matching was included in the model because layout changes were possible in the representation control condition only. All predictor variables were effect coded prior to analysis. In the models, we included a random intercept for each participant, as well as a random intercept for each task. The regression models were conducted using R [40] and the R packages lme4 [41], afex [42] and lmerTest [43] with Type 3 errors and Satterwaite approximation for degrees of freedom.

## Results

### Response time

**Fig 2:**
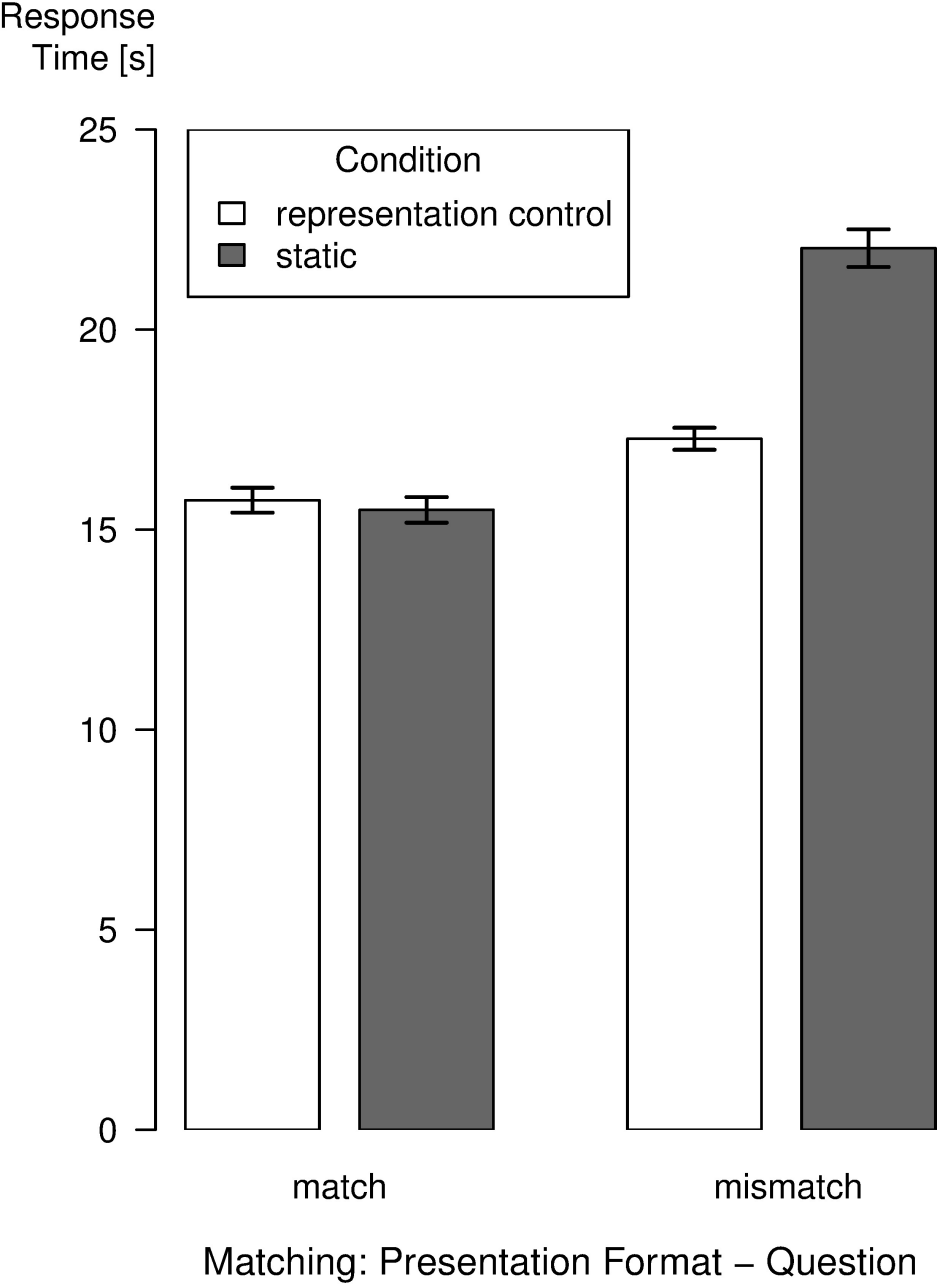
Mean response times for conditions as a function of matching between presentation format and question. Error bars represent standard errors.

The mean RTs for the representation control and the static condition across match and mismatch trials are depicted in Fig 2. The LME analysis revealed a significant interaction of matching and condition, χ^2^(1) = 81.71, *p* < .001. Planned *t*-tests with Tukey adjustments for multiple comparisons confirmed that the RTs were faster in the representation control condition than in the static condition in mismatch trials only, *t*(102.90) = -4.57, *p* < .001, whereas there was no difference between the two conditions in the match trials, *t*(100.06) = 0.19, *p* = 0.851. Furthermore, the LME revealed a significant main effect for matching, χ^2^(1) = 31.27, *p* < .001 as well as a main effect for condition, χ^2^(1) = 5.06, *p* = .025.

### Error rate

**Fig 3:**
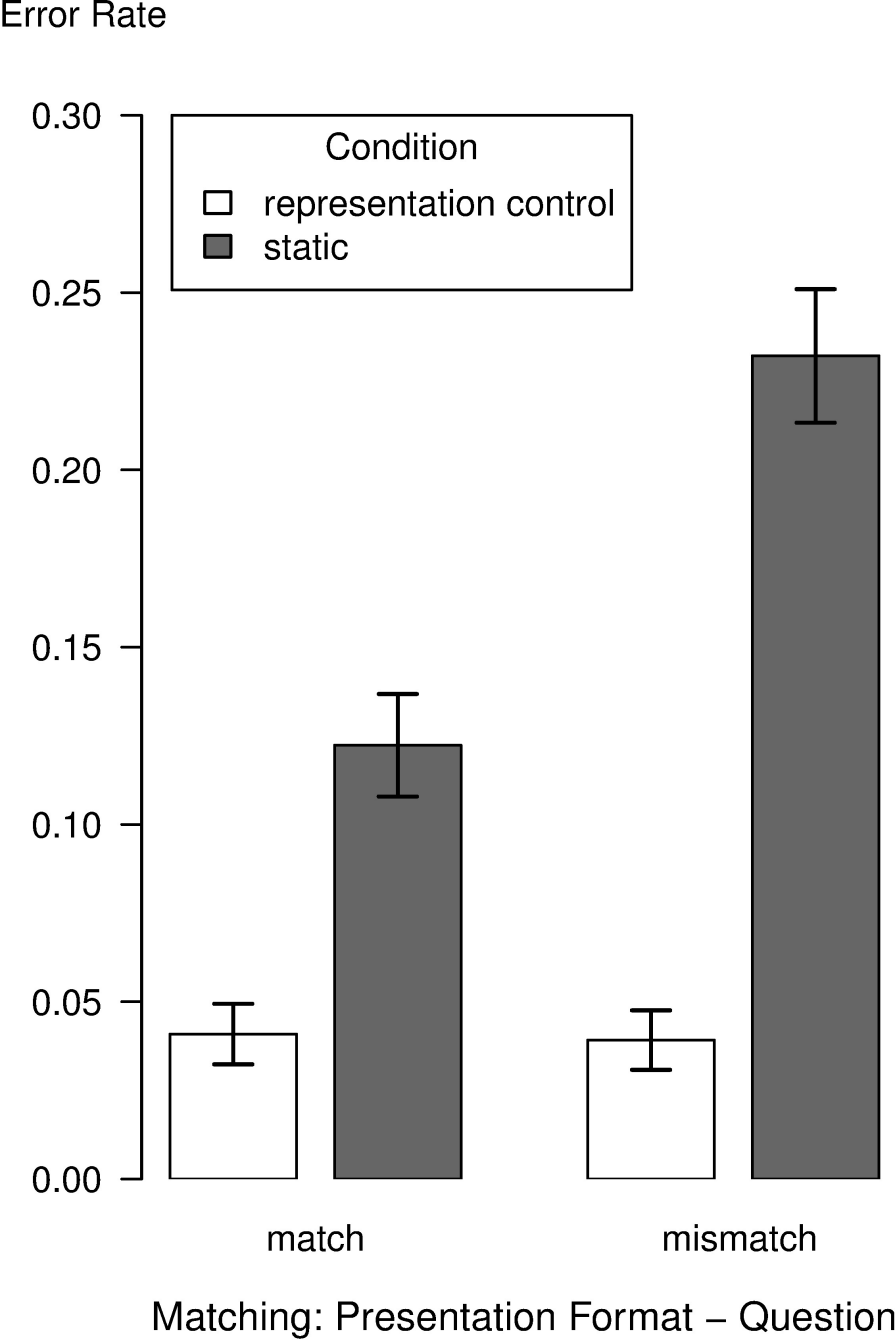
Mean error rates for conditions as a function of matching between presentation format and question. Error bars represent standard errors.

As depicted in Fig 3, error rates for the representation control and the static condition showed a similar pattern as the RTs. A GLME on ER revealed a significant interaction between the variables condition and matching, χ^2^(1) = 8.15, *p* = .004. The representation control condition and the static condition differed in mismatch trials, *z* = 6.70, *p* < .001, and also in match trials, *z* = 3.55, *p* < .001. Additionally, there was a main effect of condition, χ^2^(1) = 33.78, *p* < .001, whereas the main effect for matching did not reach significance, χ^2^(1) = 0.91, *p* = .340.

### Layout changes in representation control condition

**Fig 4:**
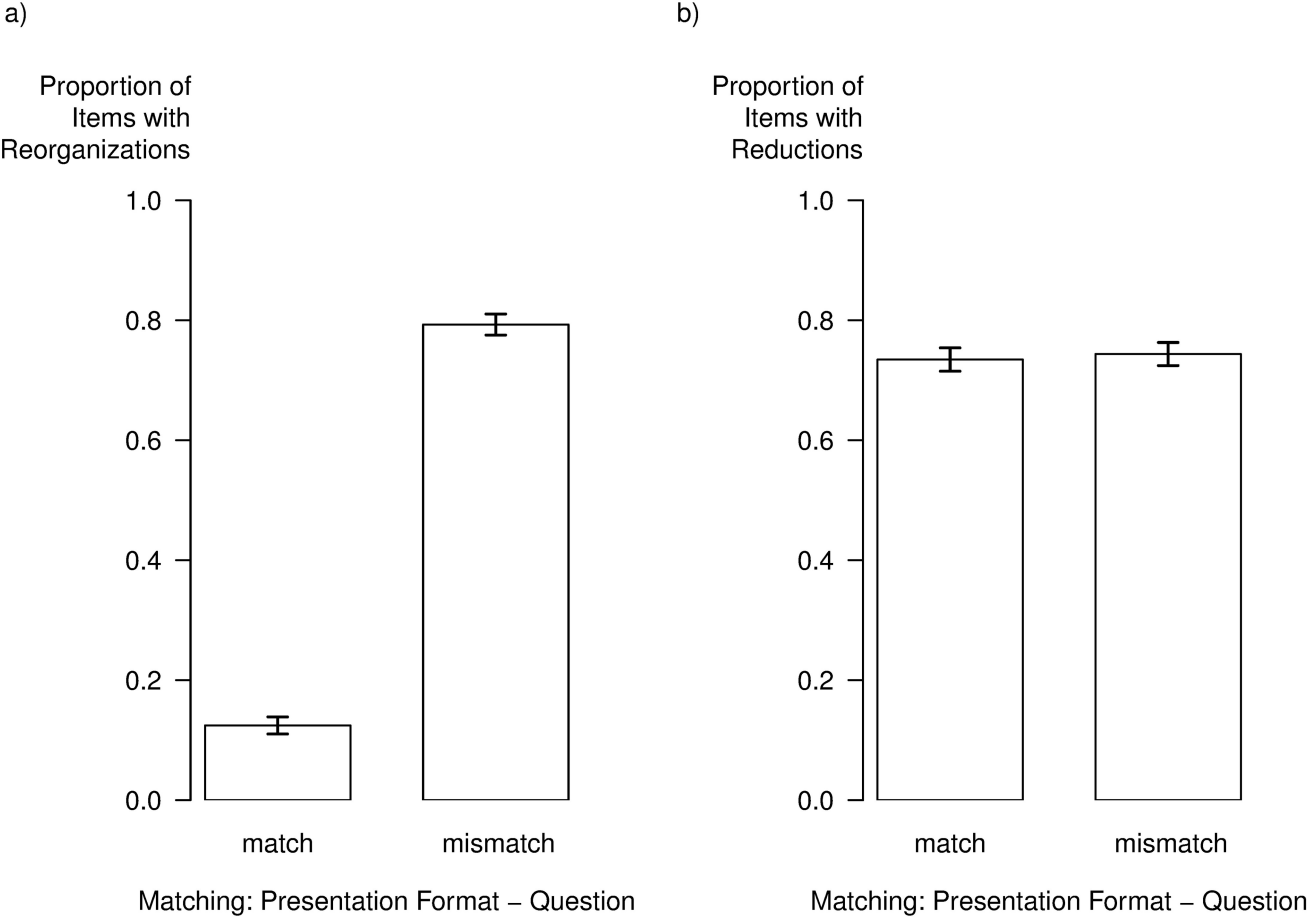
**Proportion of items with a) spatial reorganizations and b) reduction of information density as a function of matching between presentation format and question in the representation control condition.** Error bars represent standard errors.

Beyond RTs and ERs, we analyzed how frequently participants adjusted the spatial organization and information density in the representation control condition. Particularly, we were interested in whether these interactions differ for match and mismatch trials. For this purpose, we dichotomously coded whether the participants had adjusted the spatial organization of the graphical representation and/or the information density. The results are depicted in Fig 4.

Regarding the spatial reorganization, a GLME analysis revealed a significant effect of match vs. mismatch trials, χ^2^(1) = 66.57, *p* < .001. As depicted in Fig 4A, the graphical representations were reorganized in only 12% of the match trials, but in 80% of the mismatch trials. Regarding information reduction, the analysis revealed no main effect of matching, χ^2^(1) = 0.76, *p* = .383. The information density of the graphical representations was reduced equally often in match trials (73%) and in mismatch trials (74%; Fig 4B).

## Discussion

The present experiment investigated whether task performance benefits from representation control in complex graphical representations. In line with our hypothesis, participants who were able to alter the spatial organization of the presented information as well as to reduce information density of the graphical representation outperformed participants in the static group with respect to response time and accuracy. The beneficial effect of representation control was particularly pronounced in mismatch trials in which the relevant information for the question at hand was distributed across both maps. This is in line with our hypothesis that representation control is more beneficial in mismatch trials than in match trials as the option to reorganize the infographic is only useful in mismatch trials. Therefore, in mismatch trials, the participants could benefit from both transformations: the option to reduce information density and to reorganize the information, the former being in accordance with the coherence principle [44] and the latter in accordance with the spatial contiguity principle [44]. A more detailed analysis of the participants´ behavior within the representation control condition further corroborated this impression. As hypothesized, we observed reorganization of the depicted information more often in mismatch trials than in match trials. Thus, reorganization was used to adjust the spatial organization of information to the task requirements. In contrast, we observed reductions of the information density equally often in match and mismatch trials. This corroborates our hypothesis that reducing information density is useful in both types of trials. The high rate of reductions (>70%) is in accordance with former empirical results that demonstrated a benefit of sparse designs that only included relevant information [37], but contradicts the assumption of a preference for complex representations [4,38,39].

The differentiated pattern of usage of the two transformation functions of representation control–reducing information density in most match and mismatch trials and reorganizing information almost only in mismatch trials–represents a strategic, purposeful action that is executed with the aim to reduce task complexity. Consequently, participants of the representation control condition answered the questions in the mismatch trials faster and more accurately than participants in the static condition.

Our study provides evidence for the beneficial effects of the cognitive offloading of transformation processes [8]. Whereas a mental anticipation of spatial transformations is demanding with respect to mental effort, offloading such spatial transformations either in the environment [45] or into adjustments of one’s own body orientation [11] typically increases task performance. To the best of our knowledge, this is the first study that has combined the concepts of cognitive offloading and of representation control. In the present study, we could thus demonstrate that cognitive offloading does not only apply to simple tasks such as reproducing or arranging visual patterns (e.g. pattern copy tasks or Tetris games, [7,12–14], but applies also to tasks that require the integration of such visual patterns in order to form complex aggregate judgments. For such tasks, representation control allows for cognitive offloading, thereby substantially increasing task efficiency. Importantly, participants did not apply representation control in just any case but used it in a highly strategic manner. Accordingly, we were able to specify conditions under which the use of representation control is likely to occur by conceptualizing it as an instance of cognitive offloading, which is characterized by weighing the cognitive ease of processing a given visual representation against the cognitive gains of rearranging it according to the task demands. In fact, by using representation control strategically, our participants showed a high level of *meta-representational competence* [4,46,47]. This competence involves the ability to anticipate possible representations, choose the best-fitting representation, and then adjust the graph accordingly. While Hegarty and colleagues [4] focused on choosing the best representation among various representations, the present study goes one step further by giving participants the option to adjust the representations according to the task requirements. Thereby, it combines the research on representation control with the research on cognitive offloading.

Overall, the findings of the study show that digital graphical representations offer a solution to the conflict between ease of use and usefulness. Designers may include information suitable for a wide range of tasks in a graphical representation, thus increasing its usefulness. At the same time, representation control allows the viewers themselves to interactively adapt a given graphical representation to task requirements, thus increasing its ease of use. Consequently, our participants spontaneously used representation control options in order to solve the information extraction task in an efficient manner.

Note that the present study was conducted with student participants. Almost all of them reported daily use of computers. Apart from their general knowledge of digital media, they were prepared to use the representation control by an instructional video and practice trials. In Germany and German speaking countries between 75 and 80% of the population above 14 years of age are using the Internet at least sometimes [48,49]. However, it is impossible to predict the effects of interactive features for the remaining 20–25% of the population without online experience. This should be investigated in a separate study especially because recent statistics show that individuals with lower education and elderly are overrepresented within the no/low media usage group [48,49]; see also [50].

Other inter-individual differences that we did not address in the present work are ability and metacognitive knowledge. As the aim of our study was to investigate whether representation control is used in infographics in order to offload cognitive processes and to test the effects thereof, we did not measure metacognition nor the abilities of our participants. Instead, we used a rather large and homogeneous sample to minimize individual influences. However, former studies found an influence of ability and metacognitive evaluations on offloading behavior [18,16]. Risko and Gilbert [8] propose a model of the underlying processes. Future studies on the influence of task-specific abilities and metacognitive knowledge on the usage of representation control are therefore desirable.

In the present experiment, we allowed our participants to change the spatial organization and to reduce the information density. These two transformations were selected based on theoretical assumptions and allowed us to investigate the effect of representation control in a controlled experimental setup. A closer look at recent interactive infographics and maps makes clear that the options for how representation control could be implemented are manifold [51]. For example, it may allow for adding more layers of relevant information instead of eliminating unnecessary information. It may also allow for splitting a single map into several separate units instead of reorganizing relevant information into a single map. Given the close relationship of representation control with cognitive offloading that we have postulated in the present article, these types of interactive modifications should also be governed by principles of cognitive offloading. Yet, this generalization of the present findings along with its boundary conditions certainly has to be investigated in future empirical studies.

The main part of the interactive infographics that we used in the present study consisted of pairs of maps. In a recently published research agenda, Roth and his colleagues [51] identified 17 opportunities for empirical research on interactivity in maps and visualizations. Among these are included the opportunity to develop strategies to compare static and interactive maps or the opportunity to investigate the value of interactivity in new map use cases. Both are addressed in our study. Also, in visual analytics and information visualization research, interaction is an important concept [52]. Even though this field of research focuses more on knowledge construction than on information communication, some of the challenges that were identified in information visualization (e.g. capturing user intentionality) also generalize to the use of infographics for communication purposes–at least if interactive features are implemented in infographics.

The present findings demonstrate how cognitive offloading, spatial contiguity, and information coherence may inform the design of interactive knowledge media that is intended for a broad and diverse group of recipients. Particularly in online media as well as in informal educational settings in which the information goals vary across individuals, well-designed interactive features may contribute to resolve the conflict between ease of use and usefulness of complex graphical representations such as infographics or maps.
